# HIV-1 Diversity and Drug-Resistant Mutations in Infected Individuals in Changchun, China

**DOI:** 10.1371/journal.pone.0100540

**Published:** 2014-06-19

**Authors:** Ming Yan, Ke Zhao, Juan Du, Linzhang Li, Donglin Wu, Shengming Xu, Xiangchao Zeng, Guanjun Wang, Xiao-Fang Yu

**Affiliations:** 1 First Hospital of Jilin University, Changchun, Jilin, China; 2 Jilin Provincial Center for Disease Control and Prevention, Changchun, Jilin, China; 3 Johns Hopkins Bloomberg School of Public Health, Baltimore, Maryland, United States of America; Institute of Medicinal Biotechnology, Chinese Academy of Medical Sciences, China

## Abstract

**Objectives:**

Human immunodeficiency virus type 1 (HIV-1) infection has been detected in all provinces of China. Although epidemiological and phylogenetic studies have been conducted in many regions, such analyses are lacking from Jilin province in northeastern China.

**Method:**

Epidemiological and phylogenetic analyses, as well as detection of drug-resistant mutations, were conducted on 57 HIV-1 infected patients from Changchun city identified and confirmed through annual surveillance by local Centers for Disease Control in Jilin province of northeastern China in 2012.

**Results:**

Sexual contact was determined to be the major pathway for HIV-1 transmission in Jilin, where hetero- and homosexual activities contributed almost equally. Phylogenetic analyses detected multiple subtypes of HIV-1 including subtype G circulating in Jilin, with multiple origins for each of them. Both subtype B and CRF01_AE were dominant, and evidence of subtype B transmitting between different high-risk groups was observed. Mutations in the viral protease at position 71 indicated the presence of a selective pressure. Several drug-resistant mutations were detected, although they were predicted with low-level resistance to antiviral treatments.

**Conclusions:**

Information from this study fills the gap in knowledge of HIV-1 transmission in Changchun city, Jilin province, China. By revealing the origin and evolutionary status of local HIV-1 strains, this work contributes to ongoing efforts in the control and prevention of AIDS.

## Introduction

Human immunodeficiency virus type 1 (HIV-1) has continued to cause a global epidemic since its discovery in the early 1980s [1]. The prevalence of HIV-1 in China has also been increasing in recent years [2]. Although many HIV-1 surveillance studies have been conducted in different regions of China [3]–[9], only a few cases in northeastern China were reported [10], [11], and the current transmission status of HIV-1 in Jilin province remains unknown.

Several pathways are associated with HIV-1 transmission. Injection drug use (IDU) was once the dominant route for the spread of HIV-1 in China [3], [12], especially between the adjacent Yunnan and Guangxi provinces. However, recent studies revealed that sexual activities have become the major pathway for HIV-1 transmission [13]–[15], particularly among homosexual partners (or men who have sex with men, MSM) due to their secrecy, low awareness of the need for protection and difficulties in receiving intervention. In addition, infected paid blood donors (PBD) were discovered in Henan and Hubei provinces [14], [16], and subtype B HIV-1 strains were found to be highly dominant among this high-risk group throughout the country [8],[17]. Previous studies suggested that HIV-1 has been cross-transmitted among high-risk groups [18], which provides greater opportunities for the generation of circulating recombinant forms (CRF) of the virus and increases difficulties for control and prevention efforts.

In this study, an epidemiological survey of 57 HIV-1 infected individuals from Changchun city in Jilin province of northeastern China indicated that sexual contact was the major pathway for local HIV-1 transmission, while phylogenetic analyses on 35 HIV-1 strains revealed that subtype B and CRF01_AE were dominant. The sequences were also screened for drug-resistant mutations. Information gained from the observed transmission patterns among high-risk groups in this region and evidence of selective pressure may be applied towards improving treatment and prevention strategies.

## Materials and Methods

### Ethics Statement

Written informed consent was obtained from each participant involved in the study. This study was reviewed and approved by the institutional review board of the First Hospital of Jilin University, Changchun city, Jilin province, China.

### Study Subjects

HIV-1-positive subjects from Changchun city in Jilin province, China, were identified and confirmed through annual surveillance by the local Centers for Disease Control in 2012. After pretest counseling, 57 of them participated in this study with informed consent, and their personal identifiable information was encoded. Questionnaires completed by the participants provided demographic and behavioral information. All enrolled patients had previously received antiviral treatments. Blood samples were collected in 2012 and stored at −70°C.

### HIV-1 Subtyping by Polymerase Chain Reaction (PCR) and Sequencing

DNA samples corresponding to 1×10^5^ uncultured peripheral blood mononuclear cells (PBMCs) from HIV-1-positive individuals were retrieved using the DNeasy Blood & Tissue Kit (Qiagen, Valencia, CA) according to manufacturer’s protocol and subjected to PCR. The primers GAG-POL-F1, 5′-GTCCAAAATGCRAAYCCAGA-3′ (nt 1756–1775, positions corresponding to HXB2) and GAG-POL-R1 5′-TGGAGYTCATAHCCCATCCA-3′ (nt 3234–3253) were used in the first-round PCR reaction under the following conditions: 95°C for 5 min, 25 cycles at 94°C for 30 sec, 55°C for 30 sec and 72°C for 1.5 min. A sample (5 µl) of the first-round PCR product was used in the 50-µL second-round reaction mixture containing: 2.5 mM deoxyribonucleoside triphosphate, 10× PCR buffer (Mg^2+^ Plus, with 15 mM Mg^2+^), 5 U/µl Taq DNA polymerase (TaKaRa, DR100A) and 20 µM inter-nested primers for GAG-POL-F2 5′-ACAGCATGTCAGGGAGTGG-3′ (nt 1831–1849) and GAG-POL-R2 5′-ATTGCTGGTGATCCTTTCCA-3′ (nt 3006–3025). Cycling conditions were 95°C for 5 min, followed by 35 cycles at 94°C for 30 sec, 61.6°C for 30 sec and 72°C for 1.5 min.

### DNA Purification and Sequencing

PCR products were purified for sequencing by using the QIAquick PCR purification kit (Qiagen) according to the manufacturer’s protocols. Sequencing of PCR products was performed by Sangon Biotech Ltd. Co.(Shanghai, China) with an automated sequencer (PRISM automated sequencer, version 3100; ABI, Foster City, CA). For an internal control, a random selection of 25% of the samples was re-amplified, sequenced and analyzed. Identical sequences were retrieved from the control group and compared to previous sequencing results.

### Alignment and Phylogenetic Analysis

DNA alignments were performed by the ClustalW method under MEGA5 [19], followed by manual adjustment. Reference sequences were retrieved from the Los Alamos National Laboratory HIV Sequence Database (http://www.hiv.lanl.gov). Phylogenetic analysis was also conducted with MEGA5. Neighbor-joining trees were constructed using a Kimura 2-parameter model and tested by the bootstrap method with 1,000 replicates. Bootscanning analysis was also performed with Simplot 3.5.1 [20] to detect potential recombination events.

### Drug Resistance Analysis

All Gag/Pol protein sequences were submitted to the HIV Drug Resistance Database at Stanford University (http://hivdb.stanford.edu/) to detect potential resistant mutations against anti-HIV drugs.

## Results

### Study Population

In this study, 57 HIV-1-positive patients, including 47 males and 10 females from Changchun city in Jilin province, China, were identified and confirmed through annual surveillance by local Centers for Disease Control in 2012. Among all 57 patients, 27 were infected through MSM, 23 through heterosexual activity, 6 through PBD and 1 through IDU. Thus, sexual activity (87.72% in total) was the major pathway for HIV-1 transmission in Changchun. Unlike in southern cities, IDU did not contribute significantly to HIV-1 transmission, which was similar to other northern areas [10].

### Phylogenetic Analysis of Jilin HIV-1 Strains

**Figure 1:**
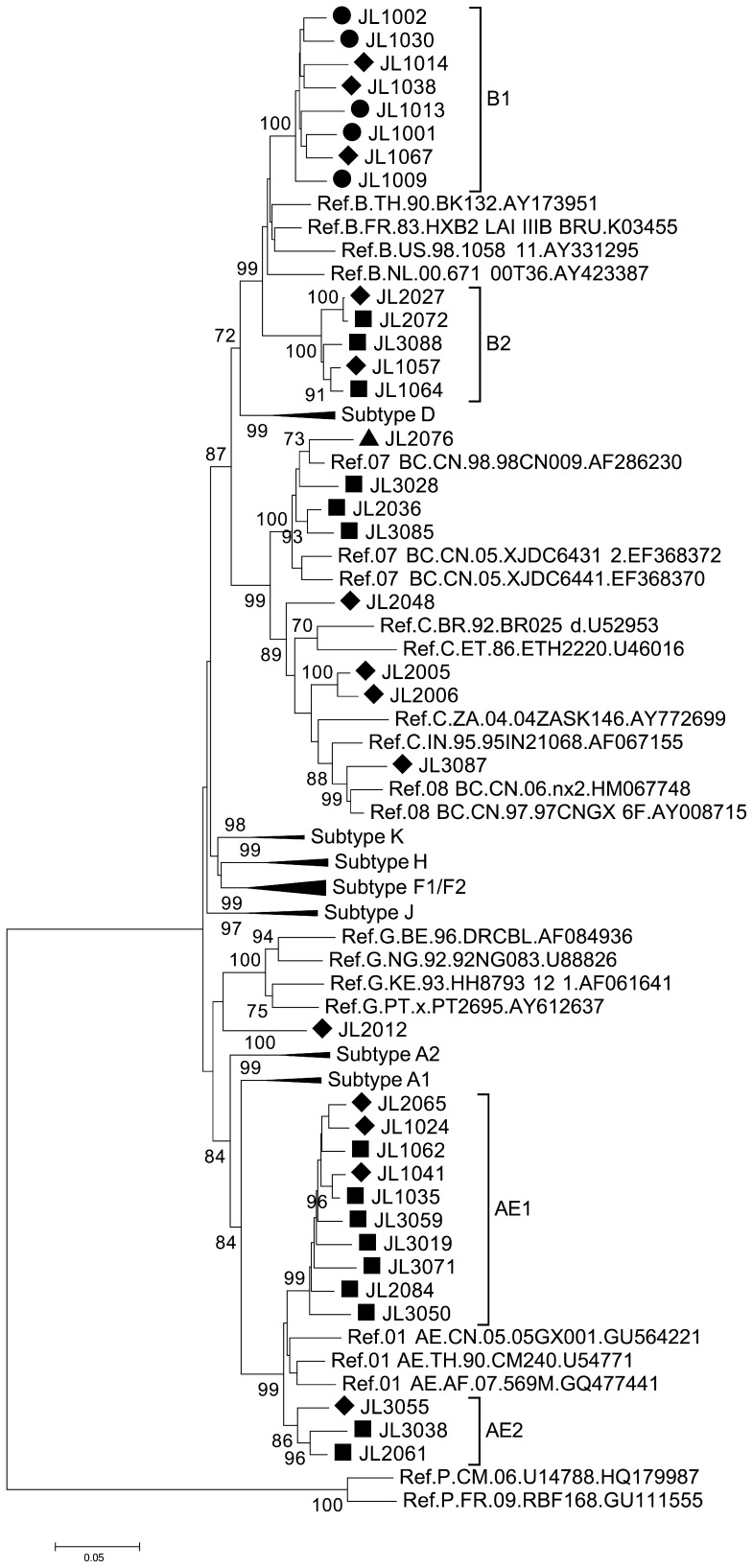
Phylogenetic analysis of *gag/pol* region (HXB2 1831–3025) of HIV-1 samples from Jilin province. The tree was constructed with MEGA5, using the Kimura-2 parameter model and the bootstrap method with 1,000 replicates. Only values above 70% are shown. Symbols indicate transmission through heterosexual activity (♦), homosexual activity (▪), blood donation (•) or IDU (▴).

Overall, 35 HIV-1 *gag/pol* sequences (positions 1831–3025 corresponding to HXB2) were successfully retrieved from the 57 samples. Phylogenetic analysis of these amplified *gag/pol* sequences revealed several subtypes circulating in Changchun (Figure 1). Subtype B and CRF01_AE were both dominant, each with the same ratio of 37.14% (13 out of 35) (Table 1). Other HIV-1 subtypes were also detected in Changchun (Figure 1 and Table 1). Four sequences were identified as CRF07_BC, three as subtype C and one as subtype CRF08_BC, leaving sequence JL2012 confirmed as subtype G.

**Table 1:** Demographic information and virus classification for HIV-1 positive patients from Changchun city*.

Patient ID	Gender	Age	Transmission Pathway	HIV-1 Subtype
JL1001	Male	33	Blood donation	B
JL1002	Male	37	Blood donation	B
JL1009	Female	37	Blood donation	B
JL1013	Male	46	Blood donation	B
JL1014	Male	36	Heterosexual activity	B
JL1024	Male	39	Heterosexual activity	CRF01_AE
JL1030	Female	30	Blood donation	B
JL1035	Male	37	Homosexual activity	CRF01_AE
JL1038	Female	42	Heterosexual activity	B
JL1041	Male	42	Heterosexual activity	CRF01_AE
JL1057	Male	34	Heterosexual activity	B
JL1062	Male	52	Homosexual activity	CRF01_AE
JL1064	Male	34	Homosexual activity	B
JL1067	Female	36	Heterosexual activity	B
JL2005	Male	35	Heterosexual activity	C**
JL2006	Female	27	Heterosexual activity	C**
JL2012	Female	29	Heterosexual activity	G
JL2027	Male	41	Heterosexual activity	B
JL2036	Male	54	Homosexual activity	CRF07_BC
JL2048	Male	29	Heterosexual activity	C
JL2061	Male	57	Homosexual activity	CRF01_AE
JL2065	Male	34	Heterosexual activity	CRF01_AE
JL2072	Male	35	Homosexual activity	B
JL2076	Male	23	Injection drug uses	CRF07_BC
JL2084	Male	48	Homosexual activity	CRF01_AE
JL3019	Male	38	Homosexual activity	CRF01_AE
JL3028	Male	38	Homosexual activity	CRF07_BC
JL3038	Male	52	Homosexual activity	CRF01_AE
JL3050	Male	44	Homosexual activity	CRF01_AE
JL3055	Female	34	Heterosexual activity	CRF01_AE
JL3059	Male	39	Homosexual activity	CRF01_AE
JL3071	Male	35	Homosexual activity	CRF01_AE
JL3085	Male	45	Homosexual activity	CRF07_BC
JL3087	Male	35	Heterosexual activity	CRF08_BC
JL3088	Male	54	Homosexual activity	B

*Only patients with positive *gag/pol* sequences are listed.

**JL2005 and JL2006 were later determined as CRF61_BC.

### Origin Analysis for each HIV-1 Subtype in Changchun City, Jilin Province

From the phylogenetic analysis, subtype B strains formed two clusters, B1 and B2, with 100% bootstrap value for each (Figure 1). All HIV-1 strains retrieved from PBDs were clustered in B1, suggesting that a transmission pathway still exists among this high-risk group. Consistently, BLAST analysis (http://blast.ncbi.nlm.nih.gov/Blast.cgi) indicated that this cluster originated from Henan province where an outbreak of HIV-1 infection once occurred among PBDs (data not shown). However, three strains also were transmitted through heterosexual activities in this same B1 cluster, indicating the presence of HIV-1 transmission between PBDs and people infected through heterosexual contact.

Besides subtype B, CRF01_AE was the other dominant subtype in Changchun city (Figure 1). It was also separated into two clusters, AE1 and AE2, each of which indicated transmission between people participating in homo- or heterosexual activities. Since the transmission pattern was similar for AE1 and AE2 (both through sexual activities), the formation of different clusters for this one subtype possibly indicated different origins for these strains. Indeed, BLAST analysis of strains from cluster AE1 revealed a high level of similarity (>97%) with other HIV-1 sequences from the neighboring province of Liaoning, which is located to the south of Jilin province. On the other hand, analysis of AE2 strains suggested an HIV-1 origin from a more distant area in China, such as Guangxi province or even from regions abroad (i.e., Vietnam or Thailand), with a maximum identity greater than 96%.

**Figure 2:**
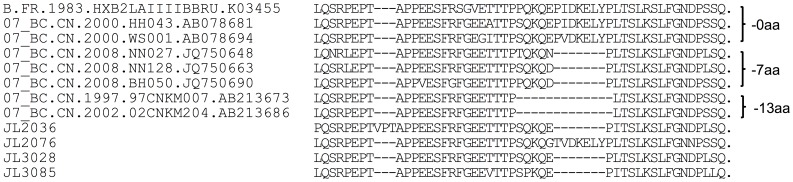
Alignment of p6 amino acid sequences retrieved from HIV-1 strains in Jilin province. The alignment was performed with MEGA5, followed by manual adjustment.

Previously, a unique in-frame deletion was found in the p6 domain of CRF07_BC sequences that was absent from CRF08_BC and CRF01_AE sequences [21], [22]. In this study, among all four *gag/pol* sequences retrieved from CRF07_BC strains in Changchun city, a 7-aa deletion (IDKELYP, according to the HXB2 sequence) could be detected in three of them (Figure 2). JL2076 as the only CRF07_BC sequence with an intact p6 region and that was transmitted through IDU, clustered with the reference sequence 98CN009, suggesting that it originated from Xinjiang province [23]. Furthermore, JL2036 and JL3085 formed a cluster with high bootstrap value (>93%) (Figure 1) and were found to originate from Beijing based on the BLAST results. JL3028 did not show a clear origin, but it was from either Beijing or Liaoning. Thus, the analysis indicated that CRF07_BC was introduced to Jilin province from multiple sources.

**Figure 3:**
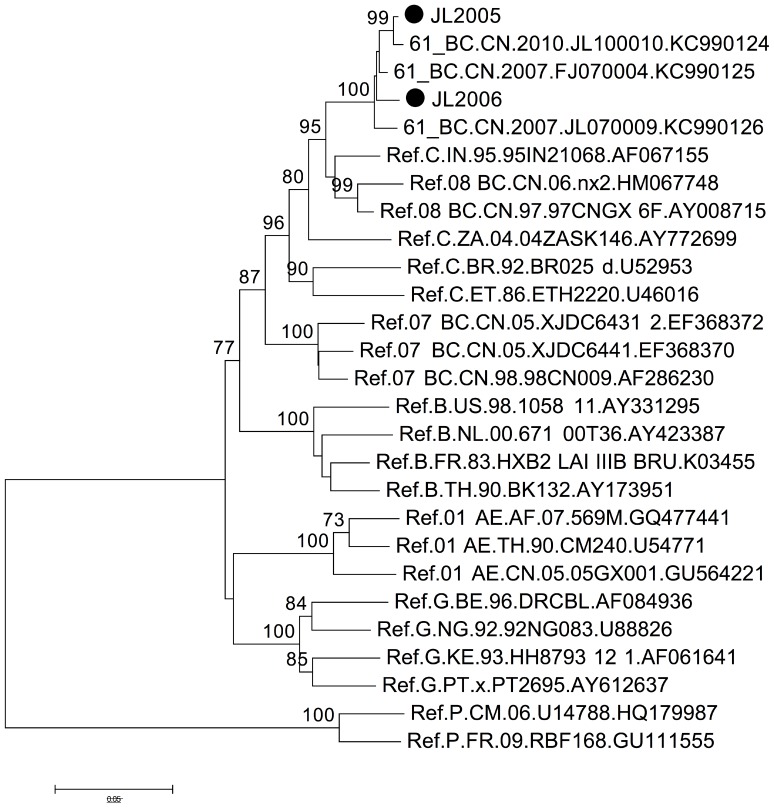
Phylogenetic analysis of *gag/pol* region (HXB2 2283–3025) of JL2005 and JL2006 from Jilin province. The tree was constructed with MEGA5, using the Kimura-2 parameter model and bootstrap method with 1,000 replicates. Only values above 70% are shown. JL2005 and JL2006 are assigned the symbol •. Besides subtype P as the outlier, only CRF61_BC reference sequences and detected HIV-1 subtypes in Jilin (Figure 1) are included.

Interestingly, JL2048 (subtype C) was found to originate from Yunnan, where HIV-1 subtype C is currently no longer dominant and rarely detected. However, BLAST results of JL2005 and JL2006, which were classified as subtype C during the initial phylogenetic analysis, showed extremely high similarity (>98% identity) to recently reported sequences of CRF61_BC [24]. The classification of JL2005 and JL2006 as this newly emerged circulating recombinant form CRF61_BC was further confirmed with phylogenetic tree construction (Figure 3).

**Figure 4:**
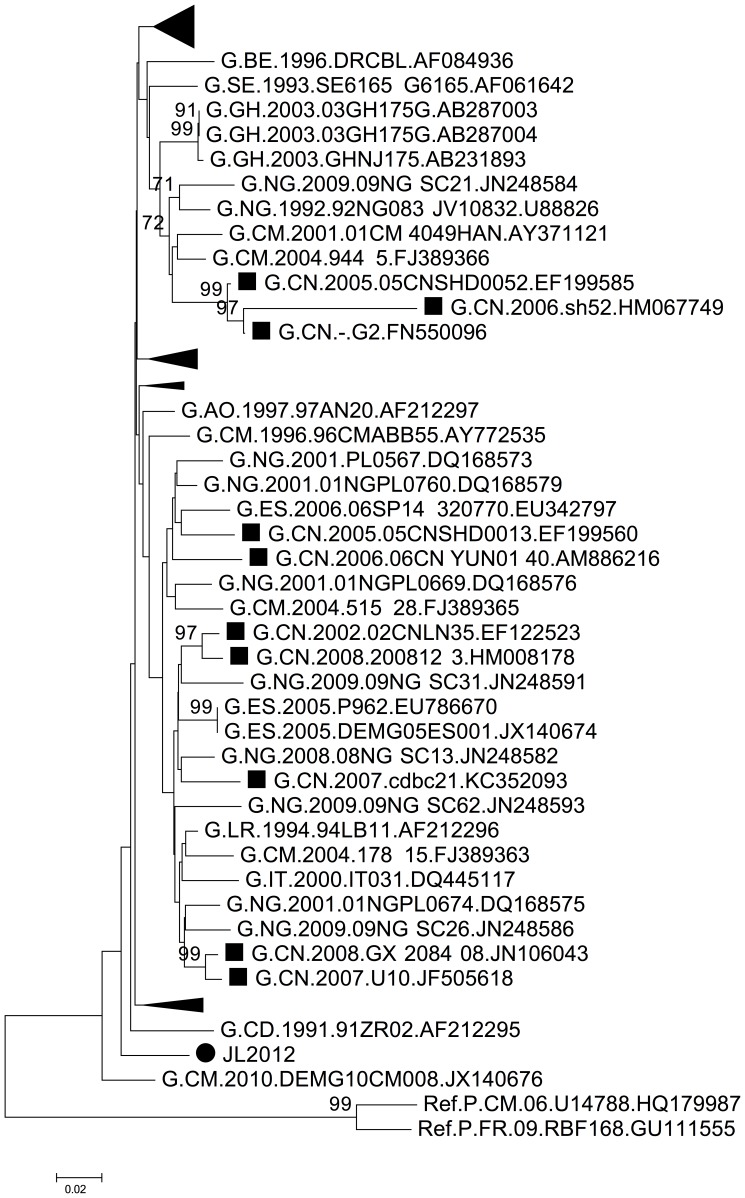
Phylogenetic analysis of *gag/pol* region (HXB2 2283–3025) of HIV-1 samples from Jilin province. The tree was constructed with MEGA5, using the Kimura-2 parameter model and bootstrap method with 1,000 replicates. Only values above 70% are shown. JL2012 is assigned the symbol •, while other subtype G strains from China are indicated with ▪. Clusters with no HIV-1 strains from China are compressed.

Several subtype G strains previously were detected in the provinces of Yunnan, Shandong, Guangdong, Guangxi and Liaoning [25]–[28]. However, BLAST analysis of JL2012 showed 95% similarity with multiple sequences from Liberia and Ghana, and only 89% with the one sequence from Liaoning province, which is adjacent to Jilin province. Phylogenetic analysis with subtype G sequences retrieved from GenBank also confirmed that JL2012 had no closer relationship with other Chinese subtype G strains (Figure 4).

### Analysis of Drug-resistant Mutations

All patients enrolled in this study had received antiviral treatments. Therefore, all sequenced *gag/pol* sequences were submitted to the HIV Drug Resistance Database at Stanford University (http://hivdb.stanford.edu/) to detect potential mutations conferring resistance against anti-HIV drugs. In total, ten retrieved sequences (28.57%) were detected with possible drug-resistant mutations against either protease inhibitors (PI) or reverse transcriptase (RT) inhibitors (Table 2). Six sequences were detected with possible PI resistance mutations, five of which occurred at position 71 of the viral protease sequence and led to no obvious drug resistance; meanwhile, one sequence had a replacement of V10 with I, which is associated with low level resistance to nelfinavir. As for RT resistance mutations, two sequences were confirmed with mutations such as A62V or K70R against nucleoside reverse transcriptase inhibitors (NRTI), while several non-NRTI (NNRTI) resistance mutations were detected. JL2048 and JL3087 contained mutations at E138 of the viral RT, leading to drug resistance of varying degrees. Notably, due to the undetermined nucleotide, JL1009 contains a mutation K101DEHN, which is highly selected by NNRTI treatment. Other detected mutations, such as V106I and K103Q, are selected by some antiviral drugs, yet no drug resistance has been linked with these mutations.

**Table 2:** Antiviral drug resistance mutations detected in HIV-1 samples from Changchun.

Patient ID	Anti-HIV-1 Drug Uses	Antiviral Drug Resistance Mutations		
		PI	NRTI	NNRTI
JL1001	D4T, 3TC, NVP	A71V		
JL1002	D4T, DDI, NVP			V106I
JL1009	D4T, 3TC, NVP	A71T	A62V	K101DEHN
JL1013	AZT, 3TC, EFV	A71T	K70R	
JL1014	D4T, DDI, NVP			K103Q
JL1038	D4T, 3TC, NVP	A71T		
JL1067	D4T, DDI, EFV	A71V		
JL2012	D4T, DDI, NVP	L10I		
JL2048	D4T, DDI, NVP			E138G
JL3087	D4T, 3TC, NVP			E138A

## Discussion

**Figure 5:**
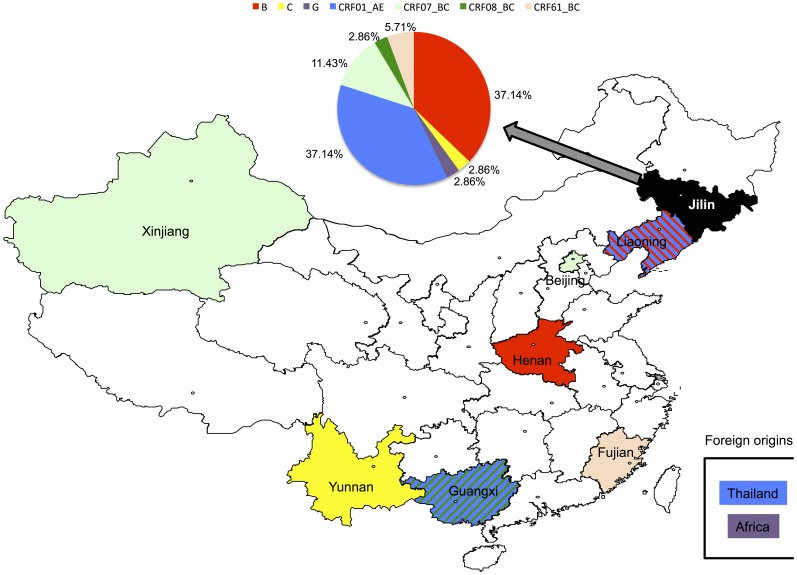
Origins of HIV-1 circulating in Jilin Province. The pie chart shows the distribution of HIV-1 subtypes circulating in Jilin province, which are color-coded above the chart. The same colors are also used on the map to indicate possible origins of HIV-1 in Jilin. Striped lines are used to indicate Liaoning and Guangxi provinces, each of which contributed two different HIV-1 subtypes to Jilin.

Due to the low rate of HIV-1 transmission in the region studied, only 57 HIV-1 patients from Changchun city were involved in this study. Nevertheless, it provides for the first time important information on the HIV-1 epidemiological pattern within the area of Jilin province. In addition to the most common subtype B in northern China, several other subtypes such as C, G, CRF01_AE, CRF07_BC, CRF08_BC and even the novel CRF61_BC were detected among the HIV-1 samples collected in northeastern China. The predominant subtypes in Changchun city were B and CRF01_AE, which are different from those of a recent HIV-1 prevalence study in Heilongjiang province in the north of Jilin [10], but highly similar to viruses from Liaoning province in the south [11]. Surprisingly, multiple origins of some HIV-1 subtypes in Changchun were observed (Figure 5): subtype B appeared to have originated in Henan and Liaoning, while subtype CRF01_AE came from Liaoning, Guangxi and even foreign countries such as Thailand. Multiple origins were also detected for CRF07_BC viruses. In addition, multiple pathways of transmission, such as through heterosexual activity, MSM, PBD and IDU, were found responsible for HIV-1 circulating in Changchun city, further increasing difficulties in prevention and control.

The geographical migration of HIV-1 in China was further demonstrated by the finding of Yunnan, where subtype C viruses are now rarely isolated, as the origin of the subtype C strain JL2048 from Jilin province. The re-classification of the two strains of JL2005 and JL2006 during our analysis from subtype C to CRF61_BC also suggested that circulating recombinant forms are replacing subtype C viruses which were once dominant in China.

HIV-1 subtype G strains are not commonly detected in China, as only 20 samples have been classified in China over the past 10 years (based on searches of the GenBank database). However, this subtype can be more easily detected in Africa, Europe and South America [29]–[31]. As indicated by both BLAST searches and phylogenetic analysis in the current study, JL2012 apparently originated directly from Africa and not within China even though subtype G strain was once detected in the neighboring province of Liaoning. As patient JL2012 had not traveled abroad, this subtype G strain potentially was imported through travelers or labor import, suggesting that increased attention is needed for monitoring international HIV-1 transmissions.

Viral DNA integrated into the human genome provides the template for replication and thus is responsible for passing on mutations, including those conferring antiviral drug resistance, to the next generation of HIV-1 virions. Therefore, we screened all Gag/Pol sequences from this study for antiviral drug resistance through the HIV Drug Resistance Database at Stanford University. All samples were retrieved from patients who had been treated with antivirals for one to three months. NRTI- and NNRTI-resistant mutations were expected to be found, as inhibitors targeting viral RT but not viral protease are widely used as anti-HIV-1 drugs in China. Indeed, we observed seven such mutations in six sequences which may be the result of selective pressure from antiviral drugs. JL1009 was possibly resistant to NNTRIs such as efavirenz (EFV), etravirine (ETR), nevirapine (NVP) and rilpivirine (RPV). However, this finding is inconclusive since multiple possible mutations were detected at position 101 on the reverse transcribed sequence of JL1009. JL2048 was predicted to have resistance against ETR and RPV. Notably, an intermediate level of resistance against zidovudine (AZT), along with low-level resistance against stavudine (D4T), was observed in the JL1013 sequence. Since this patient JL1013 was using AZT, these findings indicated that an adjustment in his antiviral therapy was required.

Although no PIs were used during prior treatment of the patients, six samples were detected with mutations in protease, leading to PI resistance of various degrees. Interestingly, five of these sequences contained a mutation at position 71, and all of these five sequences were located in the B1 cluster. Furthermore, A71T/V polymorphisms are expected at the frequency of 2∼3% without selection, but the observed ratio was 14.29% (5 out of 35), indicating the existence of additional selective pressure. Another unusual phenomenon was that sequences such as JL1009 contained multiple nucleotide mutations, which could result in potential but still undetermined drug resistance. Nevertheless, all of these observations revealed that the strains were under selection by anti-viral drugs or other factors such as local cellular tropism. As the existence of such mutations certainly presents difficulties in providing effective antiviral treatment, further confirmation of the drug resistance of these strains should be obtained in order to better control the infection in patients or prevent transmission.
